# Development of Loop-Mediated Isothermal Amplification Method for Rapid and Sensitive Identification of *Hermetia illucens* (Diptera: Stratiomyidae)

**DOI:** 10.3390/mps6050081

**Published:** 2023-09-05

**Authors:** Wenchao Zhu, Mubasher Hussain, Jing Gao, Runqian Mao, Xincheng An

**Affiliations:** 1 Guangdong Key Laboratory of Animal Conservation and Resource Utilization, Guangdong Public Laboratory of Wild Animal Conservation and Utilization, Guangdong Engineering Research Center for Mineral Oil Pesticides, Institute of Zoology, Guangdong Academy of Sciences, Guangzhou 510260, China; 2 Yantai Academy of Agricultural Sciences, Yantai 265500, China

**Keywords:** Black Soldier Fly, bioconversion, loop-mediated isothermal amplification, organic waste

## Abstract

The black soldier fly (BSF) is well known for its ability to biologically convert organic waste into insect biomass, including protein and oil, which can be utilised as animal feed. Since raw BSF products, such as BSF powder, are difficult to differentiate from other biological raw materials, therefore new analytical approaches are required. In this study, we have developed a new and fast method based on loop-mediated isothermal AMPlification (LAMP) reaction that can diagnose black soldier fly larvae and BSF byproducts with high accuracy, specificity and sensitivity. Species-specific primers for BSF were designed based on targeting the mitochondrial cytochrome C oxidase I (COI) gene. The assay was able to detect as low as 820 fg/L of BSF DNA in 60 min at 65 °C, which was a hundredfold higher than the detection limit of classical polymerase chain reaction and did not show cross-reactivity. In conclusion, the LAMP assay demonstrated excellent sensitivity and specificity to detect BSF and BSF byproducts, with a sampling-to-result identification time of 60 min.

## 1. Introduction

*Hermetia illucens* (Linnaeus), often known as the black soldier fly, is a wasp-like fly that primarily occurs in temperate and tropical climates [1,2]. The Black Soldier Fly (BSF), which originated in the South American Savanna region, has recently moved rapidly throughout Asia, Europe, Australia, and Africa [3,4,5,6,7]. BSFs have raised concerns throughout the world about their potential use in garbage management and bioconversion [8,9]. Black soldier fly larvae (BSFL), which are fed on organic waste, are utilised as a feed additive in the breading industry [10,11,12,13]. Additionally, there is a progressive increase in BSFL development in different fields. A number of facilities were set up in the south of China by entrepreneurs and the government to raise BSF and reduce hog dung [14,15]. BSFL have recently been utilised to reduce human food waste [16] and produce bio-fuel and sugar [17].

Crude byproducts of dried BSFL come to the market as a high-quality chemical raw material for extracting protein, fat, and fibre. Because of BSFL’s identical physical features (in comparison to *Tenebrio molitor* and *Zophobas atratus*, which are also employed as feed additives and chemical raw materials on the market), traditional morphological identification has been challenging to distinguish crude products of BSFL from other byproducts. Molecular techniques have long been utilised in species identification because they are more accurate than morphological and chemical detection methods. DNA level identification is required to distinguish BSF from other species.

The loop-mediated isothermal amplification (LAMP) technique is the best one for amplifying DNA because it is fast, user-friendly (requires fewer processes and reagents), portable (can be used in situ), and robust (less prone to inhibitors; therefore, it may be used with unpurified samples) [18,19,20,21,22,23,24,25]. Loop-mediated isothermal amplification (LAMP) is also a new approach for DNA amplification and has become one of the most attractive and rapidly applied technologies in the nucleic acid detection technologies field at present. According to six different sections of the target gene, four different primers were designed. Under the action of the strand-displacing DNA polymerase, Bst DNA polymerase, which is necessary for the reaction, the LAMP process can be finished within 60 min at a constant temperature instead of a complicated thermal denaturation process. LAMP technology has been widely used in human disease diagnosis, plant pathology diagnosis, food safety inspection, and other fields due to its effectiveness, speed, and high specificity. At present, researchers can use a variety of molecular biology methods to identify bio-materials, for example, restriction fragment length polymorphism (RFLP), random amplified polymorphic DNA (RAPD), and inter-simple sequence repeat (ISSR); however, these methods based on polymerase chain reaction (PCR), which is a relatively complicated process. The LAMP method can also solve the problems in conventional PCR, such as expensive equipment and complicated procedures reactions. For the fast detection of crude biological products, the LAMP procedure is a better alternative than standard PCR-based assays [26,27,28].

LAMP enables the rapid and highly precise amplification of DNA in an isothermal condition utilising DNA polymerase with strand-displacement activity [26,27,28,29]. In this study, we have used the LAMP assay method to develop a BSF-specific LAMP test that can detect both BSF and its crude products.

## 2. Materials and Methods

### 2.1. Insect Samples

**Figure 1:**
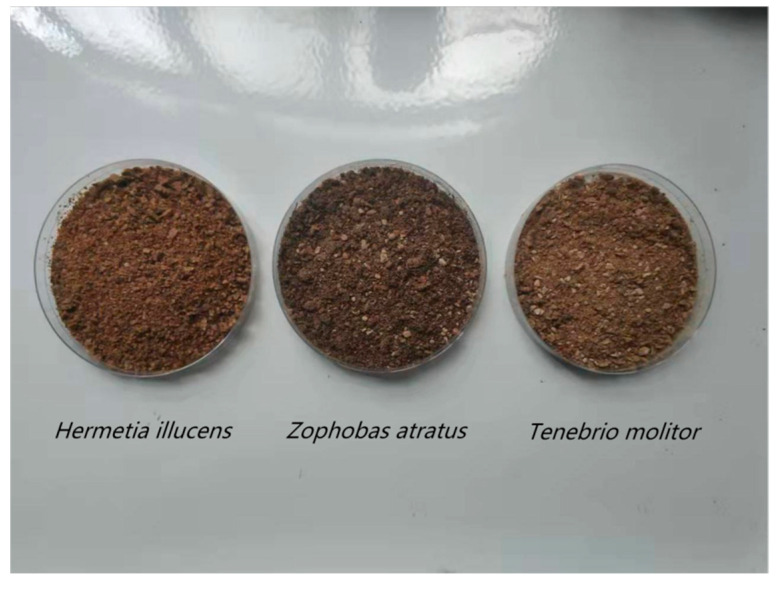
Samples of insect powder used in this study.

The black soldier fly stock population was reared in 25 °C, 16 h photoperiod, and 60–70% relative humidity feed for 30 generations at the Institute of Zoology, Guangdong Academy of Sciences, Haizhu, Guangzhou 510260, China. Fresh insects and powder of *Hermetia illucens, Zophobas atratus* and *Tenebrio molitor* (Figure 1) were purchased commercially (https://www.taobao.com, accessed on 29 August 2023) (Table 1).

### 2.2. DNA Extraction and Template Preparation

Insect fresh DNA was extracted using the Tiangen Genomic DNA Extraction Kit (DP304, Tiangen Biotech Co., Ltd., Beijing, China), and insect powder was extracted using the Ezup Food Genomic DNA Extraction Kit (B518264, Sangon Biotech Co, Ltd., Shanghai, China). COI sequences were recovered, and the origin identification was validated. Thermo Fisher Scientific’s BioMate 3 (Thermo Fisher Scientific, Waltham, MA, USA) was used to calculate the total DNA concentrations. Each of the sample DNA was stocked at −20 °C before use.

### 2.3. Design of LAMP Assay Primers

To design the LAMP assay primers, we used the reference COI sequence of the black soldier fly gene available on the National Centre for Biotechnology Information (NCBI) website (NC_035232.1, obtained from NCBI, https://www.ncbi.nlm.nih.gov/, accessed on 6 March 2021). We used a software program called Primer Explorer V5 to design a total of six primers based on six regions of the COI gene of BSF, including two outer primers, two inner primers, and two loop primers. The MEGA 9.0 software was used to align sequences. In subsequent experiments, we used a universal primer set (HCO2198, LCO1490) [30,31,32] that was designed to be specific to the BSF gene. Table 2 provides details about the primers used in the study.

### 2.4. Primer Design

**Figure 2:**
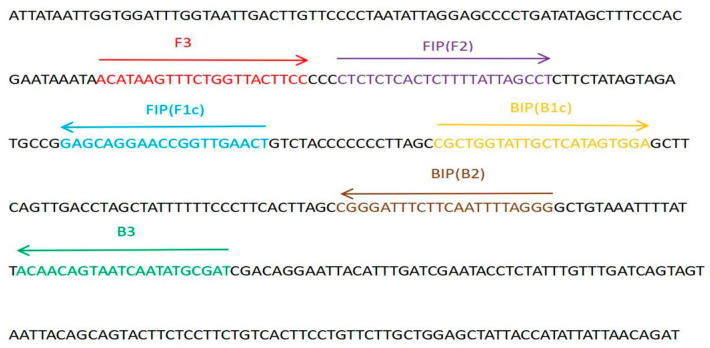
Partial COI gene sequence of BSF and location of design for LAMP primer set 35. FIP is a hybrid primer consisting of the F1c sequence and the F2 sequence, and BIP is a hybrid primer consisting of the B1c sequence and the B2 sequence. Arrows indicate the extension direction.

**Figure 3:**
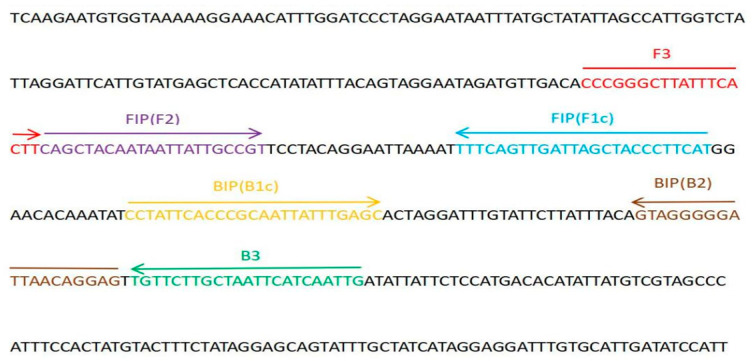
Partial COI gene sequence of BSF and location of design for LAMP primer set 109. FIP is a hybrid primer consisting of the F1c sequence and the F2 sequence, and BIP is a hybrid primer consisting of the B1c sequence and the B2 sequence. Arrows indicate the extension direction.

LAMP primers were designed based on the COI sequence of BSF (NC_035232.1, downloaded from NCBI, https://www.ncbi.nlm.nih.gov/, accessed on 29 August 2023). LAMP primers were generated using the Primer Explorer V5 program (http://primerexplorer.jp/e/index.html, accessed on 29 August 2023) (Figure 2 and Figure 3). Universal primers set (HCO2198, LCO1490) [30,31,32] was used in subsequent experiments.

### 2.5. LAMP Reaction and Amplification

LAMP reaction was carried out with a total volume of 25 μL of the reaction mixture containing 1 μL of Bst DNA polymerase (B110061, Sangon Biotech Co., Ltd., Shanghai, China), 2.5 μL of 10 × Bst DNA polymerase reaction buffer, 0.2 mM of each outer primer (F3 and B3), 1.6 mM of each inner primer (FIP and BIP), 1.4 mM of dNTPs, 6 mM MgSO_4_, and 1 μL of DNA template. To complete the process, the mixture was first incubated at 95 °C for 3 min, then at 65 °C for 60 min, and lastly at 80 °C for 10 min. The reaction temperature (60 °C, 61 °C, 62 °C, 63 °C, 64 °C, 65 °C, and 66 °C) and reaction time (60 min) were examined to determine optimal LAMP reaction conditions, and 82.0 ng/μL of DNA concentrations were used in LAMP optimisation and specificity testing. 

### 2.6. PCR Reaction and Amplification

The BSF COI sequences were also utilised to compare with the conventional PCR method. The 25 μL PCR reaction included 12.5 μL of Taq PCR Mix (B532081, Sangon Biotech Co., Ltd., Shanghai, China), 1 μL of DNA template, 1 μL of each primer (Table 1), 7.5 μL of reaction mix, and 0.4 μL of DNA polymerase. The PCR cycle processes were as follows: 3 min at 94 °C; 35 cycles of 30 s at 95 °C, 30s at 55 °C, and 45s at 72 °C; and a 7-min extension at 72 °C. 1.5% agarose gel electrophoresis was used to detect the PCR amplification products.

### 2.7. Limit of Detection of LAMP Assays

To assess the detection limit, we developed two independent methods to detect LAMP products, such as SYBR Green I dye (Invitrogen, Waltham, MA, USA) and electrophoresis on 1.5% agarose gel (six replicates were used). After adding 1 μL of SYBR Green I dye (Invitrogen, USA), the LAMP products were identified by observing the colour change with the naked eye in the first method, while electrophoresis on 1.5% agarose gels was used to assess the LAMP results in the second approach.

For the purpose of determining the sensitivity of LAMP, the measured concentration of BSF genomic DNA was diluted into a series of samples with varying concentrations (82.0 ng/μL to 82.0 fg/μL).

## 3. Results

### 3.1. Optimization of LAMP Reaction Conditions

**Figure 4:**
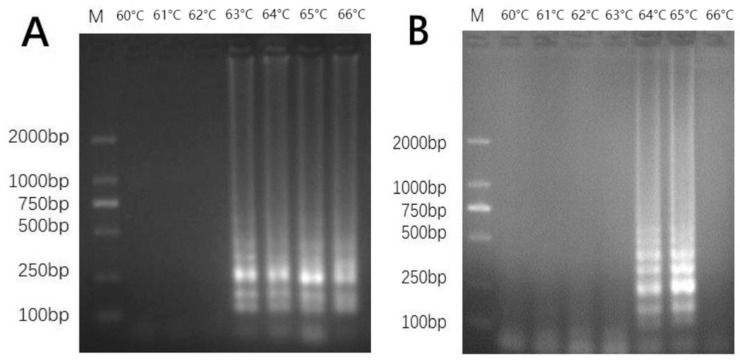
Optimization of temperature in LAMP. (**A**) primer set 35; lane 1, 60 °C; lane 2, 61 °C; lane 3, 62 °C; lane 4, 63 °C; lane 5, 64 °C; lane 6, 65 °C; lane 7, 66 °C; (**B**) primer set 109; lane 1, 60 °C; lane 2, 61 °C; lane 3, 62 °C; lane 4, 63 °C; lane 5, 64 °C; lane 6, 65 °C; lane 7, 66 °C; Lane M, 2000-bp ladder size marker.

To optimize the LAMP reaction conditions (six replicates were used), different reaction temperatures (60–66 °C) were set up. According to the results, the reactions for primer set 35 ranged in temperature from 63 °C to 66 °C; at lower temperatures (60, 61, and 62 °C), it was difficult to detect amplification, and 65 °C was the ideal temperature for the formation of the distinct ladder-like bands (Figure 4A). Similar to the previous findings, the primers set 109 reaction temperatures ranging from 64 °C to 65 °C; when the temperature was 60 °C, 61 °C, 62 °C, 63 °C, and 66 °C, there was no amplification, and 65 °C was discovered to be the ideal temperature for emerging the clearest ladder-like bands (Figure 4B).

### 3.2. Specificity of LAMP Assay

**Figure 5:**
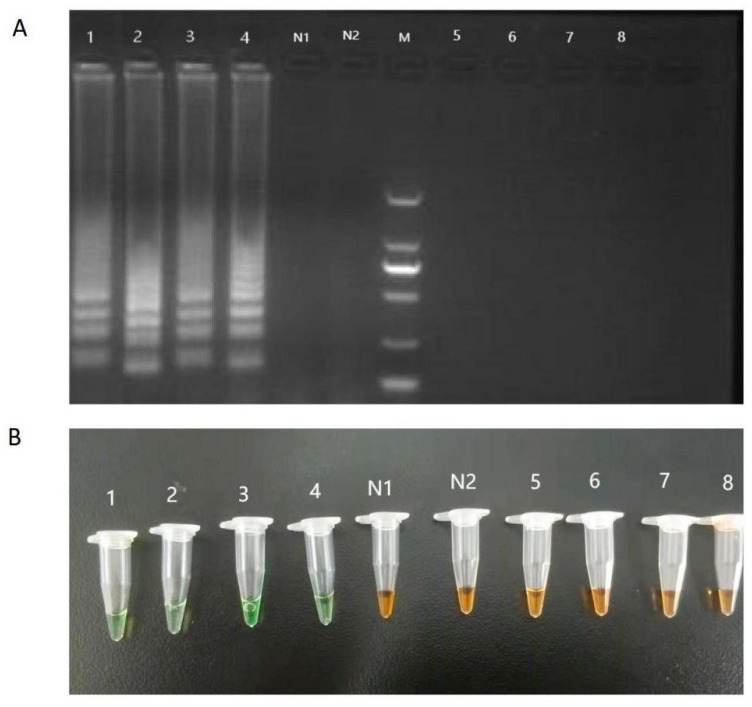
Results of specificity tests for BSF LAMP assay. Electrophoresis (**A**) and visualization (**B**) for LAMP products detection. Lanes and tubes: 1, genomic DNA of fresh BSF for primer set 35; 2, genomic DNA of fresh BSF for primer set 109; 3, genomic DNA of BSF powder for primer set 35; 4, genomic DNA of BSF powder for primer set 109; 5, genomic DNA of *Tenebrio molitor* powder for primer set 35; 6, genomic DNA of *Tenebrio molitor* powder for primer set 109; 7, genomic DNA of *Zophobas atratus* powder for primer set 35; 8, genomic DNA of *Zophobas atratus* powder for primer set 109; Lane M, 2000-bp ladder size marker; lane N1, no template for primer set 35, control (ddH_2_O); lane N2, no template for primer set 109, control (ddH_2_O).

All genomic DNA from fresh BSF was correctly detected as positive using the LAMP primer set designed for BSF powder. Our findings demonstrated that in positive samples, ladder-like bands in electrophoresis gels appeared, but no amplification was seen in negative samples (Figure 5A). The positive samples turned green under natural light after the SYBR Green I dye was added to the tubes, whereas the negative samples and tubes used as the control without a template stayed orange (Figure 5B). Assay was able to detect BSF DNA also on powder samples.

### 3.3. Analytical Sensitivity of LAMP Assay

**Figure 6:**
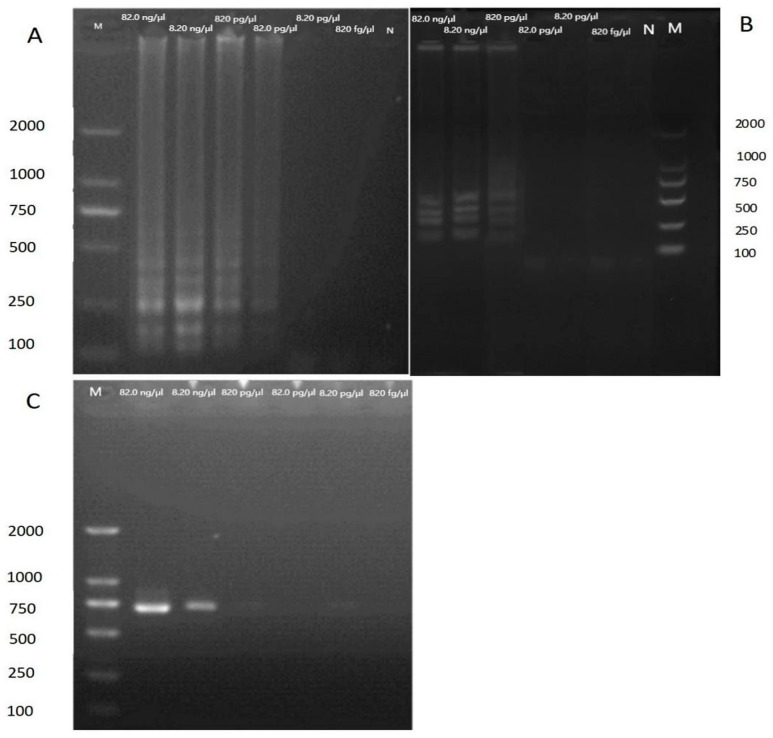
Sensitivity tests using LAMP and conventional PCR. Amplification products of LAMP primer set 35 (**A**), LAMP primer set 109 (**B**), and conventional PCR (**C**) were detected by gel electrophoresis; lane 1, 82.0 ng/μL; lane 2, 8.20 ng/μL; lane 3, 820 pg/μL; lane 4, 82.0 pg/μL; lane 5, 8.20 pg/μL; lane 6, 820 fg/μL; Lane M, 2000-bp ladder size marker; lane N, no template control (ddH_2_O).

Our results indicate that primers set 35 successfully amplified DNA concentrations ranging from 82.0 ng/μL to 8.20 pg/μL (Figure 6A), whereas primers set 109 successfully amplified DNA concentrations ranging from 82.0 ng/μL to 82.0 pg/μL (Figure 6B). Comparatively, the COI gene of BSF was amplified using conventional PCR with conventional primers; however, the detection limit of conventional PCR was only 8.20 ng/μL (Figure 6C), demonstrating that its sensitivity was 100 times lower than that of the LAMP. Therefore, LAMP was shown to be an advantageous choice for recognizing BSF due to its outstanding sensitivity.

### 3.4. LAMP Technique Application for Identifying Commercial BSF

**Figure 7:**
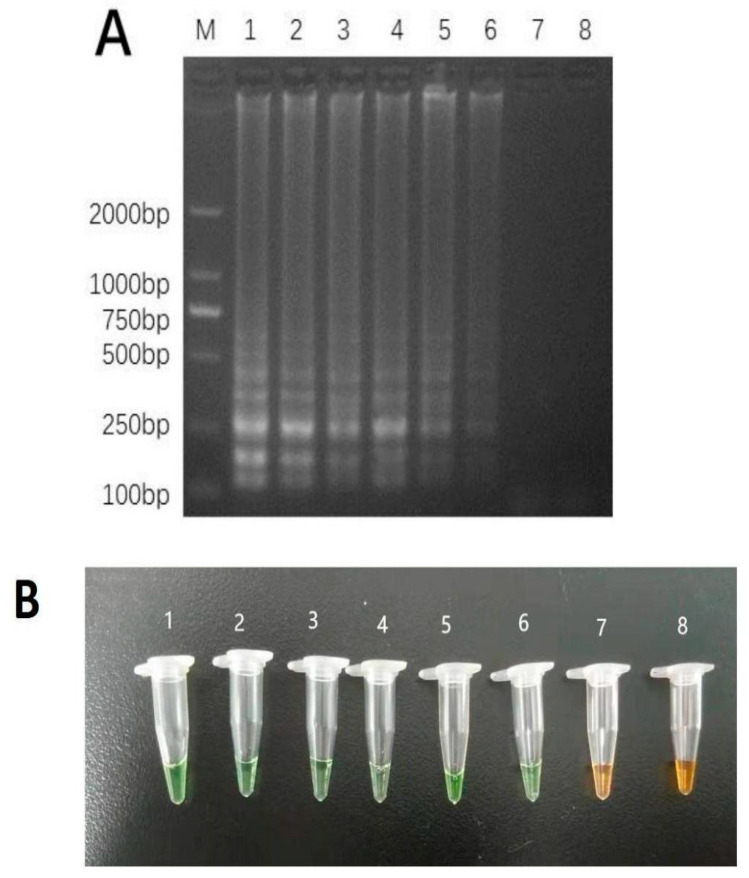
Commercial BSF product (shown in Table 1) identification using LAMP. (**A**) and visual inspection (**B**) for detection of BSF products samples from different sources; Lanes and tubes: 1, simple 7 for primer set 35; 2, simple 7 primer set 109; 3, simple 8 primer set 35; 4, simple 8 primer set 109; 5, simple 9 primer set 35; 6, simple 9 primer set 109; 7, simple 10 primer set 35; 8, simple 10 primer set 109. Lane M, 2000-bp ladder size marker.

Four samples from Taobao.com were tested (Table 1, samples 7–10) in order to further assess the reliability of the LAMP detection method we developed in the commercial field. According to the findings, the protocol was not able to detect BSF DNA in sample 10 (Figure 7).

## 4. Discussion

An accurate technique for detecting the rapid identification of insects, plants, herbal products, and their basic materials is essential in the agriculture industry. Numerous insect products are now available on the market for feed and industrial raw materials because of BSFL, and it is frequently misunderstood how to identify these insect products using standard approaches [31,32,33]. Up to now, BSFL product identification has been based on PCR-RFLP analysis, species-specific conventional PCR, or Realtime PCR. However, PCR-based methods are labor- and money-intensive, require specialized equipment to create heat cycling conditions, and are challenging to use for field testing. 

LAMP is one of the most deeply researched and well-established molecular detection approaches currently available, which provided critical support during the research and development process [34,35,36,37,38,39,40]. In comparison with the PCR-based method, the LAMP-based technique is quite easy; a heating block or water bath should suffice, and the reaction should be carried out at a steady temperature (60–65 °C). This method is particularly suited to developing countries with limited access to expensive testing equipment. Therefore, this approach has emerged as a viable alternative to PCR-based technologies not only in agricultural industry testing but also in other diagnostic assay application fields (the LAMP approach has been successfully utilized for the quick and selective detection of several types of infections) [31,32,33,34,35,36,37,38,41,42,43,44,45,46,47,48,49].

In our study, we have successfully developed a LAMP test method for rapid and accurate identification of BSF and BSFL products. DNA samples were tested using the newly designed LAMP assay, and the results were positive. We also investigated the DNA samples of BSFL products for control purposes, and none of them showed any cross-reaction with the developed assay. The developed assay was able to identify BSFL products. The high specificity of the LAMP assay for the authentication of BSFL products is attributed to the use of a set of six primers that have eight binding sites. These primers must correctly hybridize to their target gene sequence before DNA amplification can occur. This specificity ensures that the LAMP assay accurately detects BSFL products. Our results show that primers successfully amplified various DNA concentrations ranging from 820 fg/μL to 82.0 ng/μL (Figure 6A), whereas primers successfully amplified 109 different DNA concentrations ranging from 820 fg/μL to 82.0 ng/μL (Figure 6B). The COI gene of BSF was amplified using conventional PCR with typical primers, but the detection limit was only 8.20 ng/L (Figure 6C), revealing that its sensitivity was 100 times lower than that of the LAMP. As a result of its high sensitivity, LAMP was demonstrated to be an advantageous alternative for detecting BSF and BSFL products. 

Our study utilized six primers. These primers were designed based on the COI gene, which is relatively conservative and reflects the evolutionary link between species, as demonstrated by previous studies. This makes it particularly suitable for the development of BSF LAMP primers. The use of these primers results in more amplicons during the amplification phase. However, this also increases the risk of cross-contamination of following samples by other food products [50]. Despite this, the LAMP reaction can be detected faster and easier than the PCR-based method and can be applied to identify BSF and BSFL products. Our LAMP assay can, therefore, be used in the future to rapidly identify products made from plants, insects, and medicinal products.

## Figures and Tables

**Table 1:** Samples containing the target sequence.

Samples Number	Samples	Characteristics	Geographical Origin
1	BSF	Fresh	South and Central America, Australia, and Asian captive population
2	*Tenebrio molitor*	fresh	Native to Europe but spread around the world
3	*Zophobas atratus*	fresh	Tropical regions of Central and South America
4	BSF powder	processed	
5	*Tenebrio molito* powder	processed	
6	*Zophobas atratus* powder	processed	
7	BSF powder	processed	
8	BSF powder	processed	
9	BSF powder	processed	
10	BSF powder	processed	

**Table 2:** Sequences of the LAMP primers.

Amplification Method	Designation	Sequence (5′-3′)	Primer Length	Melting Temp.
LAMP	35F3-Bsf	ACATAAGTTTCTGGTTACTTCC	22	56.0 °C
	35B3-Bsf	ATCGCATATTGATTACTGTTGT	22	56.2 °C
	35FIP (Flc + F2)-Bsf	AGTTCAACCGGTTCCTGCTCCTCTCTCACTCTTTTATTAGCCT	43	56.6 °C
	109F3	CCCGGGCTTATTTCACTT	44	58.4 °C
	109B3	CAATTGATGAATTAGCAAGAACA	47	64.6 °C
	109FIP (Flc + F2)	ATGAAGGGTAGCTAATCAACTGAAACAGCTACAATAATTATTGCCGT	44	64.2 °C
	109BIP (Blc + B2)	CCTATTCACCCGCAATTATTTGAGCCTCCTGTTAATCCCCCTAC	44	64.1 °C
Conventional PCR	HCO2198	TAAACTTCAGGGTGACCAAAAAATCA	26	52.4 °C
	LCO1490	GGTCAACAAATCA AAAGATATTGG	25	52.4 °C

## Data Availability

Not applicable.
